# Revisiting stroma in pancreatic cancer

**DOI:** 10.18632/oncoscience.198

**Published:** 2015-08-21

**Authors:** Sheema Khan, Meena Jaggi, Subhash C. Chauhan

**Affiliations:** Department of Pharmaceutical Sciences and Center for Cancer Research, University of Tennessee Health Science Center, Memphis, Tennessee, USA

**Keywords:** pancreatic cancer, stroma

Clinically, pancreatic tumors have proven to develop resistance to available treatment options; however, efficacy might be achieved if the required levels of drugs reach the tumor site. Tumor microenvironment (TME) leads to extensive desmoplasia and chemo-resistance, contributing immensely to the invasive and metastatic process of pancreatic carcinoma [1]. Desmoplasia involves the excessive production of extracellular matrix and is associated with proliferation of stromal cells, including myofibroblasts and stellate cells. Recent studies suggest that the Sonic hedgehog (SHH) pathway plays a key role in desmoplasia [2] and progression of cancer, including pancreatic ductal adenocarcinoma (PDAC) [3, 4]. The implementation of stromal targeting strategies in clinical practice still poses significant challenges due to vast heterogeneity in sensitivity across different cancers and even tumor types. This may be likely due to inherent differences in expression of pro-angiogenic, invasiveness factors, growth factors/receptors and differential dependency of tumors on hypoxia and nutrition. Unlike other tumor types, pancreatic tumors that are surrounded by abundant stroma have hypo-vascularity and poor perfusion that render them less dependent on vascular supply, leading to tumor growth and hindrance in drug delivery [5]. However, so far, preclinical/clinical data reveal that despite most of the hedgehog pathway inhibitors could deplete the pancreatic tumor stroma, the outcomes outweighed over the therapeutic benefit. One plausible explanation could be the result of the side effects other than the diminution of stroma. Therefore, the identification of novel modalities that could target pancreatic stroma and overcome inaccessibility of drugs is desired.

Our study, published in Cancer Research [6], provides a preclinical proof of concept suggesting that ormeloxifene, a non-steroidal, might improve therapeutic outcomes in pancreatic cancer by targeting the stromal component. This study reinforces an insight that pancreatic tumors are responsive to stroma depletion, which might enhance the delivery of drugs to tumors. This concept was investigated in a pancreatic cancer cell line and xenograft mouse models. Ormeloxifene is a non-hormonal, non-steroidal molecule that has potent anti-cancer properties against various cancers such as breast cancer, head and neck cancer, and chronic myeloid leukemia [7]. The study demonstrates the combinatorial effects of ormeloxifene with gemcitabine at the molecular level and suggests that ormeloxifene targets the SHH signaling pathway and TME, thereby inhibiting proliferation and inducing death in PDAC cells. We observed that the effects displayed by ormeloxifene were more prominent and comparable to a known SHH inhibitor, GDC-0449 (SMO antagonist), in PDAC cells. The molecular alterations involved in the process included downregulation of SHH and its related important downstream targets such as Gli-1, SMO, PTCH1/2, NFκB-65, p-AKT and Cyclin D1. Preclinical studies revealed the ability of ormeloxifene to potentiate the anti-tumorigenic effect of gemcitabine in PDAC xenograft mice. Ormeloxifene disrupted the stroma of fibrotic pancreatic tumors and inhibited the proliferating stellate and myeloid cells that are involved in the development of fibrosis. Combination treatment with gemcitabine decreased the size of tumors and significantly inhibited metastasis. Mice treated with gemcitabine had abundant stroma, which decreased when the treatment was combined with ormeloxifene. The rich stromal component was observed in control and gemcitabine-treated mice tumor tissues, while ormeloxifene alone or in combination with gemcitabine displayed markedly less stromal component and invaded stromal tissue. This was indicated by reduced numbers of stroma myofibroblasts infiltrating the tumor tissue, as indicated by reduced PSCs, αSMA, FSP, cygb/STAP, and collagen 1 expression in the tissues. Thus, this study suggests that ormeloxifene acts by remodeling the pancreatic TME by targeting the cellular populations in the stroma that may affect tumor immune-surveillance.

**Figure 1:**
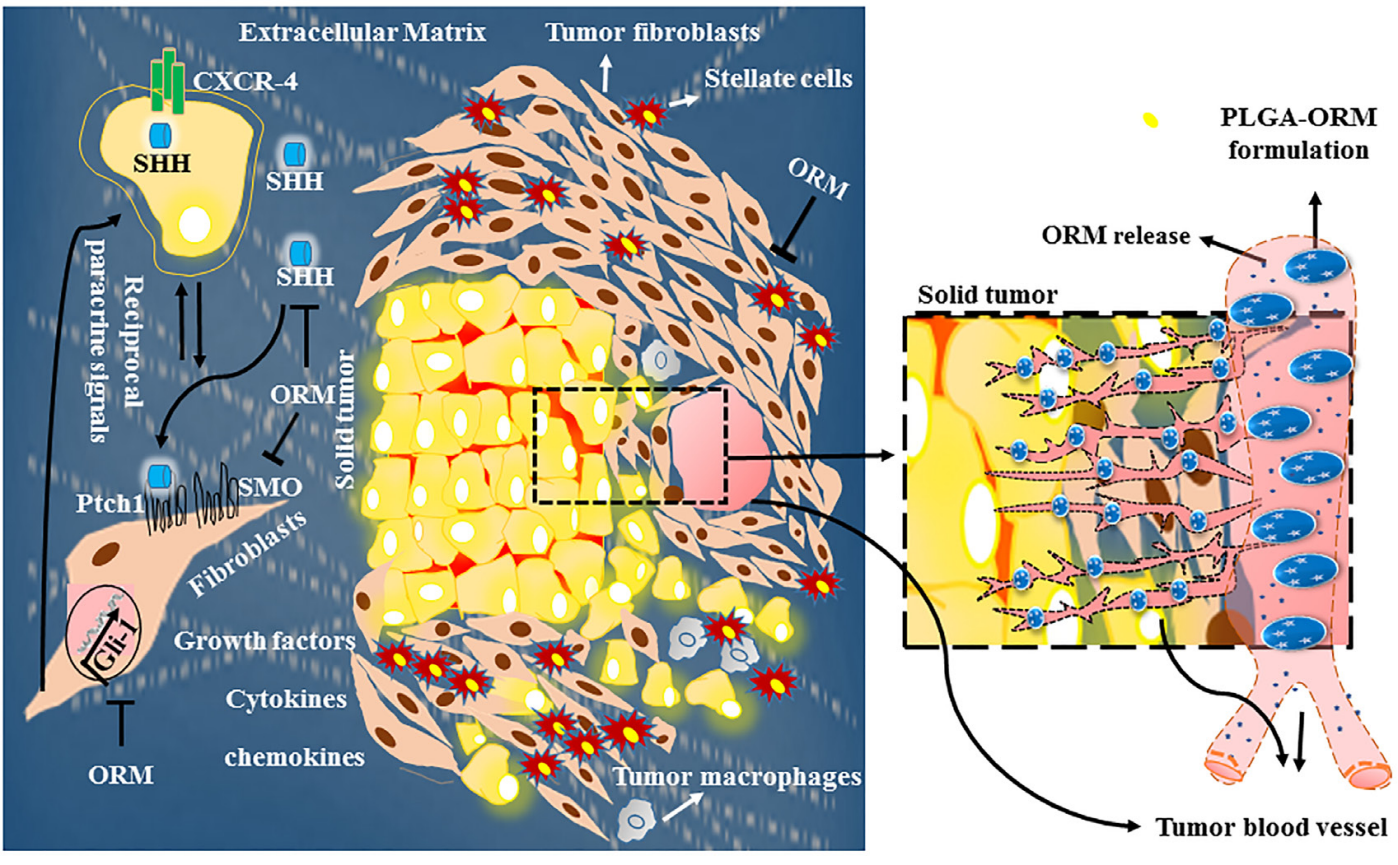
Schematic representation of the reciprocal interactions between stromal components and pancreatic ductal adenocarcinoma SHH ligand from pancreatic ductal adenocarcinoma cells stimulates stromal cells in a paracrine manner to release SMO from PTCH. The smoothened (SMO) activates Gli transcription factors, subsequently induces expression of a number of oncogenes that promote survival and proliferation of the tumor cells. ORM inhibits the SHH signaling, cross-talk between tumor-stromal components, thereby inhibiting tumor growth. Pancreatic tumors have abundant stroma and have hypo-vascularization leading to hypoxia, increased tumor growth and cause hindrance in drug delivery. ORM nanoparticle formulation displayed improved therapeutic effects in pancreatic cancer mouse model due to the accumulation and sustained release of ORM from NPs at the tumor site by the Enhanced Permeability and Retention (EPR) effect.

Considering the huge fibrotic nature of pancreatic tumors and to overcome the vast heterogeneity observed within the tumors, we have prepared a PLGA [poly(lactic-co-glycolic acid)] based nanoparticle formulation of ormeloxifene and discussed its enhanced efficacy in an article published in Biomaterials [8]. The formulation efficiently escapes lysosomal degradation and demonstrates remarkable anti-cancer potential in pancreatic cancer cells and xenograft mice. The formulation remarkably potentiated the efficacy of ormeloxifene treatment by extending median survival of the mice. Our data suggest that the formulation is highly efficient for the inhibition of pancreatic tumor growth and thus can be valuable for the treatment of pancreatic cancer. Pancreatic tumors being independent of their vascular beds and viable in hypoxic, nutrition-deprived conditions require tumor specific targeted systemic delivery of administered drugs in the absence of abundant vessels. To achieve drug specificity that can render larger accumulation in the tumor area, the formulation can be conjugated with tumor specific antibodies/aptamers (for active targeting) to preferentially reach and accumulate in tumors by both passive (Enhanced Permeability and Retention (EPR) effect) and active targeting approaches (Figure 1).

In conclusion, management of TME using this novel therapeutic modality and potential delivery system might add clinical benefit to existing standard treatments utilizing combination treatment regimens. Our studies support the understanding that manipulation of TME may overcome the stromal barrier for effective treatments in pancreatic cancer.
